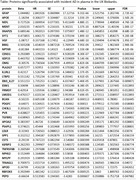# Varicella zoster and the risk of dementia: understanding the interplay between age, the genome and proteome

**DOI:** 10.1002/alz.091589

**Published:** 2025-01-09

**Authors:** Najaf Amin, M Austin Argentieri, Sihao Xiao, Cornelia M Van Duijn

**Affiliations:** ^1^ University of Oxford, Oxford, Headington United Kingdom; ^2^ Massachusetts General Hospital, Boston, MA, USA, Boston, MA USA; ^3^ Nuffield Department of Population Health, Oxford, Oxfordshire United Kingdom; ^4^ Nuffield Department of Population Health, Oxford United Kingdom; ^5^ Nuffield Department of Population Health, University of Oxford, Oxford United Kingdom

## Abstract

**Background:**

The mechanism through which common viruses such as varicella zoster virus (VZV) may trigger the onset of AD in the elderly is far from understood. The poliovirus receptor (PVR) is a protein of interest as it was associated to AD in a brain proteomics study and interacts with multiple viruses (Wingo, et al. Nat Neurosci). In this study (UKB), we examine whether plasma PVR levels are associated to AD, two of its early blood‐based biomarkers (NeFL and GFAP), ageing and VZV seropositivity.

**Methods:**

Plasma proteins including PVR have been measured in 53,000 UKB participants using the OLINK platform. In this cohort, 592 individuals developed AD during the 13 years follow‐up. For 967 participants, seropositivity for VZV is measured. We used Cox regression to associate PVR and other proteins to AD, adjusting for age, sex, physical activity, smoking, body mass index, alcohol use, 25 medications for chronic diseases, and technical covariates. We used Mendelian Randomization (MR) to test whether PVR is likely in the causal pathway based on genetic variants in the encoding genes. Further, we associated PVR to NeFL, GFAP, a protein‐based biomarker for accelerated aging (Argentieri et al, medRxiv) and seropositivity for VZV.

**Results:**

PVR was found to be one of the 37 plasma proteins that were significantly associated incident AD (false discovery rate < 0.05) (see Table). The MR experiment conducted suggests a causal association between PVR and AD. We further find that PVR is significantly associated with the two biomarkers of AD pathology studied, NeFL (p<2.00*10^−16^) as well as with GFAP (p<2.00*10^−16^). Since the gene encoding for PVR maps to the 19q31 region close to APOE, we adjusted for APOE genotypes. The association of PVR with AD, NeFL and GFAP remained significant. We further find that PVR is strongly associated to accelerated proteomic aging (p = 2.54*10^−15^) as well to VZV serum positivity (p = 0.05).

**Conclusion:**

Our findings suggests that PVR may play a role in the interplay between VZV seropositivity, age and AD. PVR may be relevant for the association between other viruses and AD.